# Recombinant Polymerase Amplification Coupled with CRISPR/Cas12a Detection System for Rapid Visual Detection of Porcine Circovirus 3

**DOI:** 10.3390/ani14172527

**Published:** 2024-08-30

**Authors:** Genghong Jiang, Xiaoyu Yang, Zhaoyang Li, Jingyu Mao, Penghui Zeng, Dedong Wang, Zhi Wu, Changzhe Liu, Yonghui Qiu, Yongqiu Cui, Jianwei Zhou, Jue Liu, Lei Hou

**Affiliations:** 1 Department of Preventive Veterinary Medicine, College of Veterinary Medicine, Yangzhou University, Yangzhou 225009, China; jiang20220827@163.com (G.J.); yxiaoyu0512@163.com (X.Y.); goaheadli2022@163.com (Z.L.); 19975069589@163.com (J.M.); zengpenghui1120@163.com (P.Z.); wddyzu@163.com (D.W.); liuchangzhe@bioprofile.cn (C.L.); 18238776375@163.com (Y.Q.); cuiyongqiu97@163.com (Y.C.); jwzhou@yzu.edu.cn (J.Z.); liujue@263.net (J.L.); 2 Jiangsu Co-Innovation Center for Prevention and Control of Important Animal Infectious Diseases and Zoonoses, Yangzhou University, Yangzhou 225009, China; 3 Loudi Livestock, Aquaculture and Agricultural Machinery Affairs Center, Loudi 417000, China; 19848049073@163.com

**Keywords:** Porcine circovirus 3 (PCV3), recombinant polymerase amplification (RPA), CRISPR/Cas12a system, one- and two-pot methods, detection

## Abstract

**Simple Summary:**

Porcine circovirus type 3 (PCV3) is a newly discovered pathogen that causes porcine dermatitis and nephrotic syndrome (PDNS)-like clinical signs. The current lack of effective vaccines and therapies for PCV3 has led to a main reliance on early diagnosis and detection for the prevention and control of PCV3 infection. Nucleic acid detection is commonly used for the early identification of pathogens, and the sensitivity and specificity of two-pot RPA-CRISPR/Casa-Ps, the rapid, effective, sensitive, and specific detection method for PCV3 is, indeed, needed. Here, we develop one- and two-pot methods with a combination of recombinant polymerase amplification (RPA) and a CRISPR/Cas12a detection system for the simple, rapid, sensitive, specific, and visual detection of PCV3 clinical samples within 30 min. These two detection methods have the potential to detect clinical PCV3 infections.

**Abstract:**

The porcine circovirus type 3 (PCV3) infection is an emerging disease associated with clinical signs of porcine dermatitis and nephropathy syndrome (PDNS)-like clinical signs. Currently, there is a lack of effective vaccines and therapeutics against this disease. Therefore, rapid, effective, sensitive, and specific detection methods are crucial for the timely identification, prevention, and control of PCV3. In this study, we developed one- and two-pot visual detection methods for PCV3 using a clustered regularly interspaced short palindromic repeat (CRISPR)/Cas12a detection system combined with recombinase polymerase amplification (RPA). These two methods demonstrated no cross-reactivity with eight other swine viruses and exhibited minimum detection limits of five and two copies of viral DNA, respectively, revealing their high specificity and sensitivity. During a clinical sample detection within 30 min, the coincidence rates between the one- and two-pot detection methods and real-time quantitative polymerase chain reaction (qPCR) were 100%. In conclusion, both one- and two-pot RPA-CRISPR/Cas12a detection methods have significant potential for the rapid, sensitive, and specific visual detection of PCV3.

## 1. Introduction

Porcine circoviruses (PCVs), belonging to the Circovirus genus of the Circoviridae family, are small, non-enveloped viruses with single-stranded circular DNA genomes [1]. Currently, PCVs are divided into four genotypes, PCV1, PCV2, PCV3, and PCV4, which have similar compositions [2]. PCV1, which originates from a contaminant in cells, is not pathogenic [3]. PCV2 is a major pathogen involved in post-weaning multisystemic wasting syndrome (PMWS) and swine dermatitis and nephrotic syndrome (PDNS) [4,5]. PCV4 infection causes respiratory diseases and diarrhea in pigs [5]. PCV3 is associated with porcine dermatitis, nephropathy syndrome (PDNS), and reproductive failure, and its genome mainly encodes a replicase protein (Rep), capsid protein (Cap), and an unknown function (ORF3) [6]. Cap protein, the only structural protein, shares approximately 37% amino acid identity with PCV2, which determines the antigenic characteristics of PCV3 [6,7].

The spread of PCV3 has caused severe economic losses in the swine industry. To date, there are no commercial vaccines or effective drugs against this disease, which makes early detection and monitoring of pathogens particularly important for preventing and controlling PCV3 infection. Serological detection mainly depends on various enzyme-linked immunosorbent assay (ELISA) formats owing to their high throughput and low cost [8,9]. A limitation of these detection methods is that the samples need to be obtained a long time after viral infection, which misses the early detection period. In contrast, nucleic acid amplification tests, such as polymerase chain reaction (PCR) and reverse transcription (RT)-PCR, can provide the fast, sensitive, and specific detection of early viral infections [10,11]. However, these detection methods commonly require specialized equipment, trained technicians, and a certain reaction time (approximately 2 h), which is not suitable for real-time detection of pathogens.

The clustered regularly interspaced short palindromic repeat (CRISPR)-associated endonuclease (CRISPR/Cas) system has been used to detect nucleic acids because of its rapidity, visualization, simplicity, and high sensitivity [12,13]. The Cas12a system targets and unwinds double-stranded DNA by recognizing the protospacer-adjacent motif (PAM) under the guidance of CRISPR RNA (crRNA) [12,14]. Next, the reporter single-stranded DNA (probe), as a readout for detection, performs nonspecific cleavage [15,16]. The Cas12a system combined with isothermal recombinant polymerase amplification (RPA) has been widely used to detect various pathogens, including African swine fever virus [17], severe fever with thrombocytopenia syndrome virus [18], Haemophilus parasuis [19], and severe acute respiratory syndrome virus 2 [20].

In this study, we developed one- and two-pot RPA-Cas12a for the visual detection of PCV3. The whole reaction of the one- and two-pot assays was completed within 30 min at 37 °C with a sensitivity of viral DNA detection under five and two copies, respectively. In addition, 25 clinical tissue samples were analyzed by both one- and two-pot RPA-Cas12a methods, in which the coincidence rate of test results with real-time quantitative PCR (qPCR) was 100%, indicating the reliability and applicability of these two methods in clinical PCV3 diagnosis.

## 2. Materials and Methods

### 2.1. Viruses, Clinical Samples, and Antibodies

PCV2, PCV3, PCV4, the porcine pseudorabies virus (PRV), Senecavirus A (SVA), and the porcine epidemic diarrhea virus (PEDV) were obtained from our laboratory. The viral genome (DNA or RNA) of PCV1, the porcine reproductive and respiratory disorder syndrome virus (PRRSV), and the classical swine fever virus (CSFV) were provided by Changchao Huan, Nanhua Chen, and Chengcheng Zhang at Yangzhou University, respectively. All clinical tissue samples, including spleen, lung, kidney, submandibular lymph node, and inguinal lymph node samples, were collected from different pig farms. Mouse anti-His antibodies (ab18184) and horseradish peroxidase (HRP)-conjugated anti-mouse secondary antibodies (A9044) were purchased from Abcam (Cambridge, UK) and Sigma-Aldrich (St. Louis, MO, USA), respectively.

### 2.2. The Expression and Purification of Cas12a Protein

A full-length fragment of Cas12a was amplified from the 6 His-MBP-TEV-huLbCas12a plasmid (#90096; Addgene, Beijing, China) and cloned into the pET-28a vector to generate the pET-28a-Cas12a expression plasmid. Following validation by sequencing, this plasmid was transformed into BL21 competent cells (DE3) (TSC-E06; Tsingke Biotech, Beijing, China) to express the Cas12a protein. Cas12a proteins were purified using a His-tag protein purification kit (DP101-01; Transgen Biotech Co., Ltd., Shenzhen, China) and analyzed by sodium dodecyl sulfate-polyacrylamide gel electrophoresis (SDS-PAGE) and Western blotting. The purified Cas12a proteins were stored at −80 °C. The pcold-TF-PCV3 Cap plasmid (PCV3 Cap DNA) was maintained in our laboratory.

### 2.3. Viral DNA or cDNA Preparation of Various Viruses and Clinical Samples 

Viral DNA was extracted from viral supernatants (PCV2, PCV3, PCV4, and PRV) and clinical samples (200 mg) in 1 mL of phosphate-buffered saline (PBS) through homogenization, freeze–thaw cycles, and centrifugation using a virus DNA purification kit (B518267-0050; Sangon Biotech, Shanghai, China). For viral cDNA preparation: total RNA was extracted from SVA or PEDV supernatants using TRIzol reagent (15596018; Invitrogen, Carlsbad, CA, USA) according to the manufacturer’s protocol, and cDNA synthesis for SVA, PEDV, PRRSV, and CSFV was performed using the Vazyme cDNA Synthesis Kit (R323-01; Vazyme, Nanjing, China).

### 2.4. Design and Synthesization of crRNAs and ssDNA

Known-target DNA (KT-DNA) and crRNA (KT-crRNA) were synthesized by GENEWIZ [21]. Three crRNAs targeting the PCV3 Cap gene (GenBank No: MF318451) were designed using online design software (https://zlab.squarespace.com/guide-design-resources (accessed on)) according to the PAM sequence (TTTN) of recognization by Cas12a and synthesized by GENEWIZ. An ssDNA probe labeled with the fluorescent group 6-FAM and quenching group BHQ-1 (ssDNA-FQ probe) was synthesized by GENEWIZ.

### 2.5. Assay of Endonuclease Activity of the Purified Cas12a Proteins and Optimization of crRNAs and Cas12a Concentration

The purified Cas12a proteins were mixed with 1 μL KT-DNA (2 × 10^9^ copies/µL), 1 μL KT-crRNA (1 μM), or 1 μL ssDNA-FQ probe (10 μM) in assay buffer (B6002V; New England Biolabs, Ipswich, MA, USA) (NEB Buffer) and then incubated at 37 °C for 30 min. The endonuclease activity of Cas12a was detected using blue, ultraviolet, and fluorescent signals (INFINITE M PLEX).

Three designed crRNAs (crRNA1, 2, or 3) were mixed with Cas12a, 1 μL viral DNA (2 × 10^9^ copies/μL), and 1 μL ssDNA-FQ probe (10 μM) in assay buffer, respectively, and then incubated at 37 °C for 30 min. The best crRNA was identified and screened based on fluorescence signals. For the optimization of Cas12a and crRNA concentration in CRISPR/Cas12a-PCV3 detection method, the various Cas12a protein concentrations (0.25, 0.5, or 1 μg/μL) and crRNA concentrations (0.25, 0.5, or 1 μM) were mixed with 1 μL PCV3 Cap DNA (2 × 10^9^ copies/μL), and 1 μL ssDNA-FQ probe (10 μM) in assay buffer, respectively, and then incubated at 37 °C for 30 min. The optimal concentrations of Cas12a and crRNA were determined based on fluorescence signals. 

### 2.6. The Optimization of Recombinant Polymerase Amplification (RPA)

Three pairs of RPA primers targeting the PCV3 Cap gene were designed using Snapgene software v7.2. The RPA reaction system was set to a 50 µL final reaction volume, including 30 μL buffer, 2.5 μL each of forward and reverse primers (10 μM), 1 μL PCV3 Cap DNA, 2.5 μL Magnesium Acetate (MgOAC, 280 nM), and appropriate sterile water. The RPA reaction was performed at 37 °C for 20 min using the TwistAmp Basic Kit (TABAS03KIT; Twist D X, Cambridge, UK), followed by 1% agarose gel electrophoresis.

### 2.7. The Optimization of Sensitivity and Time in CRISPR/Cas12a-PCV3 Detection

The copy numbers of pcold-TF-PCV3-Cap plasmid were calculated as follows: DNA copy numbers = (6.02 × 10^23^) × (DNA concentration (ng/μL) × 10^−9^)/(DNA length× 660 Da/bp). The CRISPR/Cas12a reaction system was set to a 50 µL final reaction volume including 30 μL buffer, 2.5 μL each of forward and reverse primers (10 μM), 1 μL 10-fold serially diluted PCV3 Cap DNA (concentrations from 2 × 10^9^ to 2 × 10^6^ copies/µL), 2.5 μL MgOAC (280 nM), and appropriate sterile water. The CRISPR/Cas12a reaction was performed at 37 °C for 30 min to analyze sensitivity according to the fluorescence signals.

For the optimization of reaction time in CRISPR/Cas12a-PCV3 detection, the CRISPR/Cas12a-PCV3 detection system in the two-pot method was set to a 50 μL final reaction volume including 1 μL Cas12a (0.5 ug/μL), 1 μL crRNA (0.5 μM), 2 μL ssDNA-FQ probe (10 μM), 5 μL NEB Buffer, 1 μL PCV3 Cap DNA (2 × 10^9^ copies/µL), and appropriate sterile water. CRISPR/Cas12a-PCV3 detection was performed at 37 °C, and the fluorescence signals at 5, 10, 20, 30, and 40 min were analyzed. 

### 2.8. The Analysis of Sensitivity and Specificity in Two-Pot RPA-CRISPR/Cas12a-PCV3 Detection

The RPA reaction system was set to a 50 µL final reaction volume including 30 μL buffer, 2.5 μL each of forward and reverse primers (10 μM), 1 μL 10-fold serially diluted PCV3 Cap DNA (concentrations 2 × 10^11^, 2 × 10^9^, 2 × 10^7^, 2 × 10^5^, 2 × 10^3^, 2 × 10^1^, and 2 × 10^0^ copies/µL, respectively) or 1 μL DNA or cDNA of various viruses (PCV1, PCV2, PCV3, PCV4, PRV, PRRSV, SVA, CSFV, and PEDV), 2.5 μL MgOAC (280 nM), and appropriate sterile water. The RPA reaction was performed at 37 °C for 20 min using the TwistAmp Basic Kit, and the 5 μL of reaction products were added to the CRISPR/Cas12a reaction system, followed by CRISPR/Cas12a detection to analyze sensitivity and specificity according to the fluorescence signals.

### 2.9. Establishment and Sensitivity of One-Pot RPA-CRISPR/Cas12a-PCV3 Detection Method

To simplify the procedure and reduce contamination, we adapted the RPA amplification step and the CRISPR/Cas12a-PCV3 detection step in one tube by adding the amplification reagents to the bottom of the tube and the detection reagents to the cap of the tube, followed by CRISPR/Cas12a detection.

The total reaction system was set to 40 μL final reaction volume to optimize reaction sensitivity. The total reaction volume of 40 μL included 32 μL amplification reagent (15 μL buffer, 1.5 μL each of forward and reverse primers, 1 μL viral DNA of various concentrations, 1 μL MgOAC, and 12 μL sterile water) at the bottom of the tube and 8 μL CRISPR/Cas12a-PCV3 detection reagent (1 μL Cas12a, 1 μL crRNA, 2 μL ssDNA-FQ probe, 4 μL NEB Buffer). The one-tube RPA-CRISPR/Cas12a-PCV3 detection procedure as follows: after the RPA amplification at 37 °C for 20 min, the tube was centrifuged and then incubated at 37 °C for 10 min to perform CRISPR/Cas12a-PCV3 detection.

### 2.10. The Detection of Clinical Samples

DNA was extracted from 25 clinical samples (200 mg/sample) in 1 mL PBS through grinding, freeze–thaw, and centrifugation using a viral DNA purification kit and then detected by qPCR using the reported primers [21] and one- or two-pot RPA-CRISPR/Cas12a-PCV3 detection methods. All positive samples were further validated by sequencing.

### 2.11. Statistical Analysis

Significant differences were evaluated using one-way analysis of variance (ANOVA) or Student’s *t*-test using Prism 8.0 software (GraphPad Software, Boston, MA, USA), with a *p* value < 0.05 considered statistically significant.

## 3. Results

### 3.1. Expression and Purification of Cas12a Protein and Assay of Endonuclease Activity

The Cas12a gene was amplified from the 6 His-MBP-TEV-huLbCas12a plasmid and cloned into the pET28a vector (Figure S1A). To obtain Cas12a proteins, a prokaryotic expression system was used for expression. Cas12a proteins were mainly expressed in the bacterial lysate supernatants (Figure S1B), which were purified and detected by SDS-PAGE and Western blotting (Figure S1C,D). Subsequently, the endonuclease activity of the purified Cas12a protein was assayed by ultraviolet light, blue light, and fluorescence signals at 37 °C for 30 min in an RNase-free centrifuge tube containing detection buffer, the known-target DNA (KT-DNA) and crRNA (KT-crRNA) [21], and a synthesized ssDNA-FQ probe. The results showed that both blue and ultraviolet light were positive (Figure S1E), which was consistent with the fluorescence signals (Figure S1F), suggesting that the purified Cas12a protein had good endonuclease activity.

### 3.2. The Optimization of Cas12a and crRNA Concentration in CRISPR/Cas12a-PCV3 Detection Method

Three crRNAs targeting the PCV3 Cap gene were designed and synthesized by GENEWIZ, according to the PAM sequence (TTTN) recognized by Cas12a (Figure S2A, Table S1). To screen the best crRNA, three crRNAs were assayed in the CRISPR/Cas12a-PCV3 detection method at 37 °C for 30 min, respectively. The results showed that the fluorescence signal of crRNA3 was the strongest, and so it was selected for subsequent experiments (Figure S2B,C). Next, the concentrations of Cas12a and crRNA3 were optimized in the CRISPR/Cas12a reaction based on their fluorescence signals. The results showed that the appropriate concentrations of Cas12a protein and crRNA3 were 0.5 μg/µL and 0.5 μM, respectively (Figure S2D,E).

### 3.3. The Optimization of RPA Primers and Sensitivity of CRISPR/Cas12a-PCV3 Detection

The RPA amplification and CRISPR/Cas12a-PCV3 detection stages play important roles in the RPA-CRISPR/Cas12a-PCV3 detection method. For the RPA amplification stage, three pairs of RPA primers (RPA1, RPA2, and RPA3) targeting the conserved motif of PCV3 Cap were designed and screened. The PCV3 Cap DNA was 10-fold serially diluted from 2 × 10^9^ to 2 × 10^0^ (2 × 10^9^, 2 × 10^7^, 2 × 10^5^, 2 × 10^3^, 2 × 10^1^, and 2 × 10^0^, respectively) copies/µL, and amplified by RPA for analyzing the sensitivity of RPA amplification. The results showed that the amplification sensitivity of the RPA3 primer was higher than that of the other two primers (Figure S3A). For the CRISPR/Cas12a-PCV3 detection stage, the 10-fold serially diluted viral DNA concentrations from 2 × 10^9^ to 2 × 10^6^ (2 × 10^9^, 2 × 10^8^, 2 × 10^7^, and 2 × 10^6^, respectively) copies/µL were directly measured by fluorescence signal using the CRISPR/Cas12a-PCV3 detection method. The results showed that the detection sensitivity of this method was 2 × 10^9^ copies/µL (Figure S3B,C).

### 3.4. Establishment of Two-Pot RPA-CRISPR/Cas12a-PCV3 Detection Method

**Figure 1:**
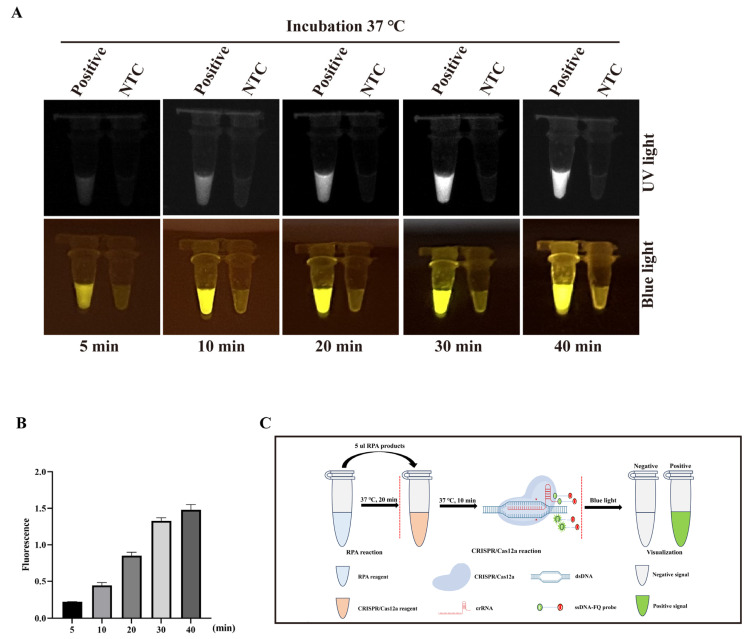
The development of the two-pot RPA-CRISPR/Cas12a-PCV3 detection method. (**A**,**B**) The measurement of the CRISPR/Cas12a reaction targeting PCV3 DNA at various times (5, 10, 20, 30 or 40 min) by blue light, UV light (**A**), and fluorescence signal (**B**). (**C**) Schematic diagram of two-pot RPA-CRISPR/Cas12a-PCV3 detection method. The data are expressed from three independent experiments.

The two-pot RPA-CRISPR/Cas12a-PCV3 detection method was established by combining RPA amplification and CRISPR/Cas12a-PCV3 detection, with further optimization of the total reaction time. The amplification time of RPA was set to 20 min, according to the RPA amplification kit. Subsequently, the various times (5, 10, 20, 30, or 40 min) were optimized in the CRISPR/Cas12a-PCV3 detection targeting PCV3 Cap DNA (2 × 10^9^ copies/µL). The results showed that the UV light, blue light, and fluorescence signal increased with time and peaked at 40 min in the CRISPR/Cas12a-PCV3 detection stage, whereas the control group did not show any positive signal (Figure 1A,B). A positive fluorescence signal was observed after 5 min; therefore, we artificially set the detection time to 10 min based on the timeliness of this detection method. Finally, the total time for this two-pot detection method was set to 30 min (RPA for 20 min and the CRISPR reaction not exceeding 10 min) (Figure 1C).

### 3.5. Sensitivity and Specificity of Two-Pot RPA-CRISPR/Cas12a-PCV3 Detection Method

**Figure 2:**
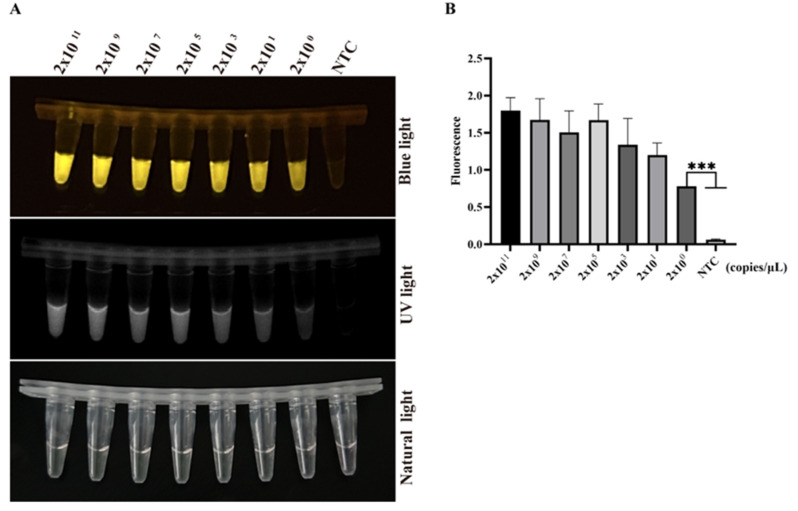
The sensitivity of two-pot RPA-CRISPR/Cas12a-PCV3 detection method. (**A**,**B**) The sensitivity of two-pot method detection with 10-fold serially diluted viral DNA (concentrations 2 × 10^11^, 2 × 10^9^, 2 × 10^7^, 2 × 10^5^, 2 × 10^3^, 2 × 10^1^, and 2 × 10^0^ copies/µL, respectively) by blue light, UV light (**A**), and fluorescence signal (**B**). The data are expressed as the means ± SDs from three independent experiments (*** *p* < 0.001).

The sensitivity of the two-pot RPA-CRISPR/Cas12a-PCV3 detection method was determined by the UV light, blue light, and fluorescence signal with or without 10-fold serially diluted PCV3 Cap DNA (concentrations 2 × 10^11^, 2 × 10^9^, 2 × 10^7^, 2 × 10^5^, 2 × 10^3^, 2 × 10^1^, and 2 × 10^0^ copies/µL, respectively). The results showed that the minimum detection limit for this method was 2 × 10^0^ copies/µL (2 copies/µL) of viral DNA (Figure 2A,B).

**Figure 3:**
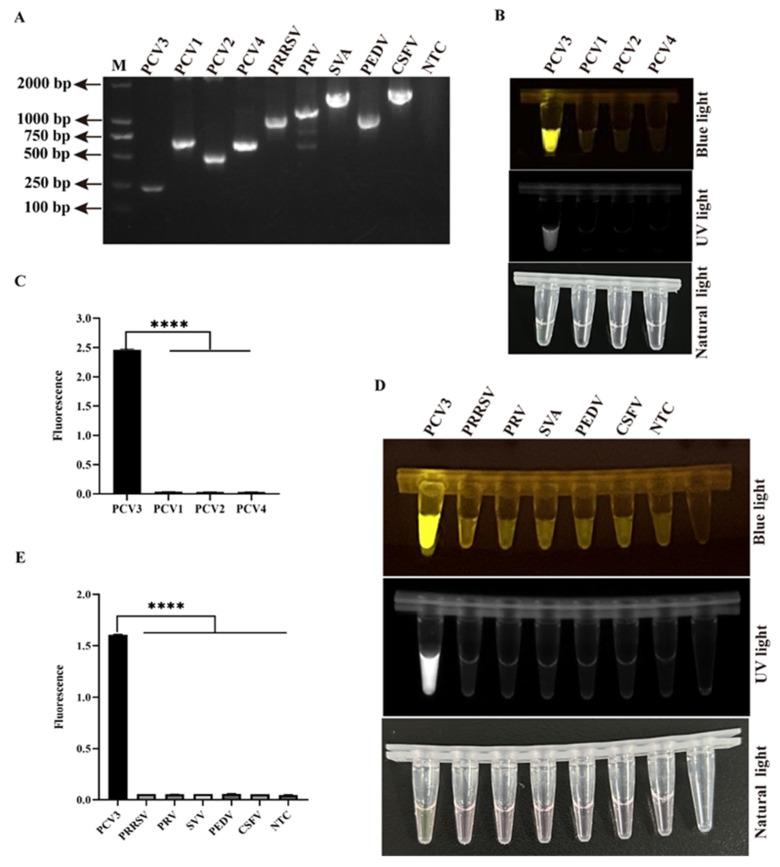
The specificity of two-pot RPA-CRISPR/Cas12a-PCV3 detection method. (**A**) The specific PCR products of some swine viruses (PCV3, PCV1, PCV2, PCV4, PRRSV, PRV, SVA, PEDV, and CSFV). (**B**,**C**) The specificity of two-pot method detection with four genotypes of PCV (PCV3, PCV1, PCV2, and PCV4) by blue light, UV light (**B**), and fluorescence signal (**C**). (**D**,**E**) The specificity of two-pot method detection with some swine viruses (PCV3, PRRSV, PRV, SVA, PEDV, and CSFV) by blue light, UV light (**D**), and fluorescence signal (**E**). NTC represents no-viral DNA or cDNA control. The data are expressed as the means ± SDs from three independent experiments (**** *p* < 0.0001).

The specificity of the two-pot RPA-CRISPR/Cas12a-PCV3 detection method was further analyzed. Viral DNA or RNA of nine swine viruses were confirmed by conventional PCR or RT-PCR (Figure 3A). Porcine circoviruses have four genotypes, namely PCV1, PCV2, PCV3, and PCV4 [2], prompting us to determine the specificity of this method for various genotypes. The extracted genomes from the four PCV genotypes were detected, and the results showed that only the PCV3 genome exhibited a positive UV light, blue light, and fluorescence signal, whereas the other viral genotypes did not (Figure 3B,C). Subsequently, the specificity of the two-pot RPA-CRISPR/Cas12a-PCV3 detection method for DNA or RNA viruses (PRV, PRRSV, SVA, PEDV, and CSFV) was analyzed. As shown in Figure 3D,E, the PCV3-positive sample, instead of the other samples, showed a specific UV light, blue light, and fluorescence signal. Together, these results indicate that the two-pot RPA-CRISPR/Cas12a-PCV3 detection method is highly sensitive and specific for PCV3.

### 3.6. Evaluating Consistency between Two-Pot RPA-CRISPR/Cas12a-PCV3 Detection Method and Real-Time Quantitative PCR (qPCR) Method

**Figure 4:**
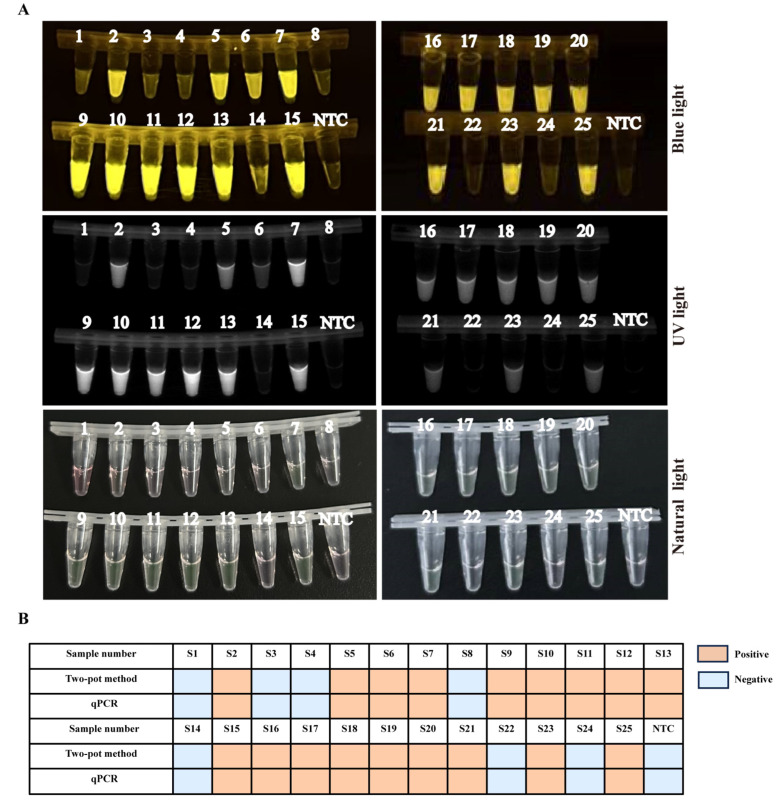
Detection of PCV3 clinical samples by two-pot RPA-CRISPR/Cas12a-PCV3 detection method. (**A**) The 25 clinical samples detection of two-pot RPA-CRISPR/Cas12a-PCV3 detection method by blue light, and UV light. (**B**) Comparison of detection results using two-pot RPA-CRISPR/Cas12a and qPCR-based PCV3 detection methods, with blue indicating negative results and orange indicating positive results. NTC represents no-viral DNA control.

To evaluate the performance of the two-pot RPA-CRISPR/Cas12a-PCV3 detection method, 25 clinical samples, including spleen, lung, kidney, submandibular lymph node, and inguinal lymph node, were analyzed. As shown in Table 1 and Figure 4A, 18 of 25 clinical samples were positive, while the remaining samples were negative. To verify the reliability of the two-pot RPA-CRISPR/Cas12a-PCV3 detection method for the detection of PCV3, a previously published qPCR method was used to verify the above detection results. The detection results of the two-pot RPA-CRISPR/Cas12a-PCV3 detection method and qPCR were consistent (Figure 4B), with a 100% coincidence rate, suggesting that the two-pot RPA-CRISPR/Cas12a-PCV3 detection method is reliable.

### 3.7. The Clinical Samples Detection of One-Pot RPA-CRISPR/Cas12a-PCV3 Method 

**Figure 5:**
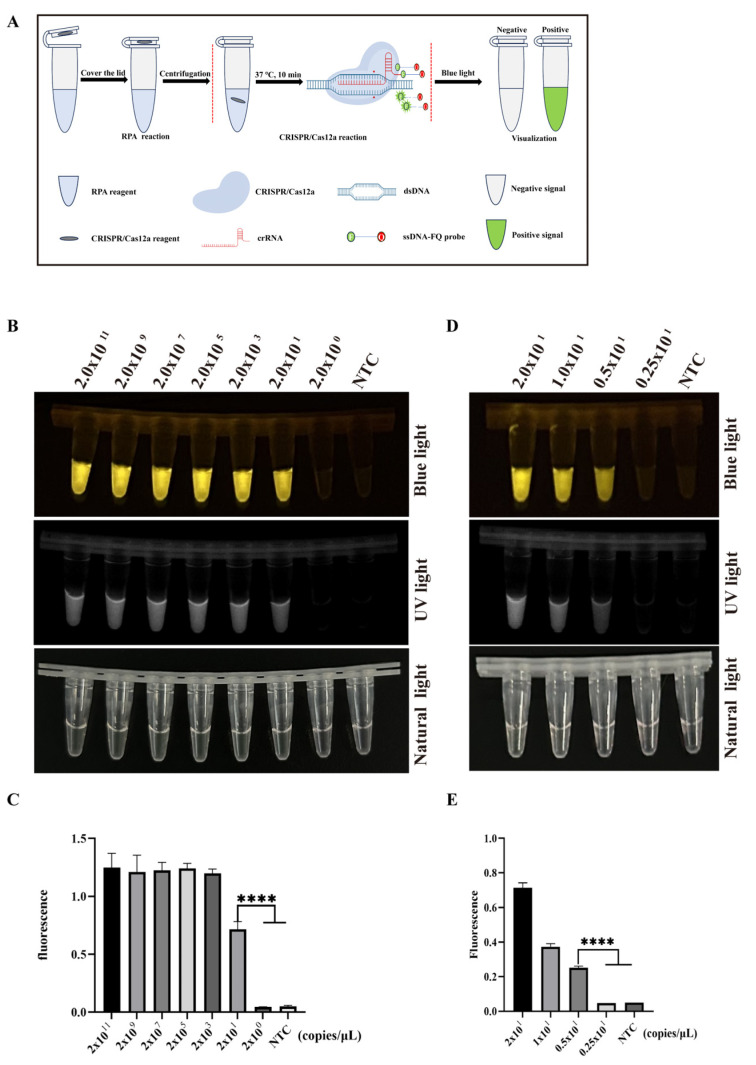
The development of one-pot RPA-CRISPR/Cas12a-PCV3 detection method and its sensitivity. (**A**) Schematic diagram of one-pot RPA-CRISPR/Cas12a-PCV3 detection method. (**B**,**C**) The sensitivity of one-pot method detection with 10-fold serially diluted viral DNA (concentrations 2 × 10^11^, 2 × 10^9^, 2 × 10^7^, 2 × 10^5^, 2 × 10^3^, 2 × 10^1^, and 2 × 10^0^ copies/µL, respectively) by blue light, UV light (**B**), and fluorescence signal (**C**). (**D**,**E**) The sensitivity of one-pot method detection with 2-fold serially diluted viral DNA (concentrations 2 × 10^1^, 1 × 10^1^, 0.5 × 10^1^, and 0.25 × 10^1^ copies/µL, respectively) by blue light, UV ligh (**D**), and fluorescence signal (**E**). The data are expressed as the means ± SDs from three independent experiments (**** *p* < 0.0001).

**Figure 6:**
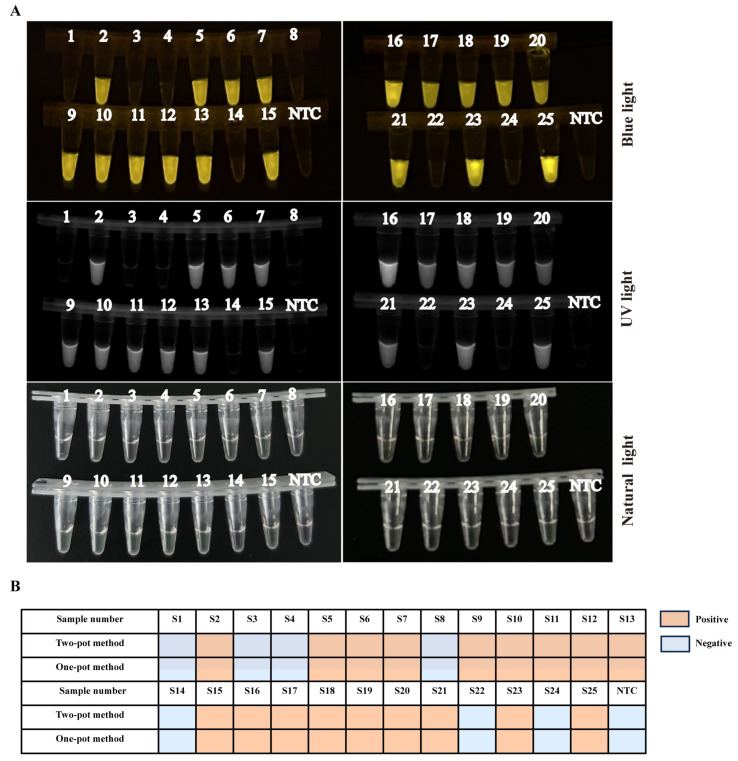
Detection of PCV3 clinical samples by one-pot RPA-CRISPR/Cas12a-PCV3 detection method. (**A**) The 25 clinical samples detection of one-pot RPA-CRISPR/Cas12a-PCV3 detection method by blue light and UV light. (**B**) Comparison of detection results between one- and two-pot RPA-CRISPR/Cas12a detection methods, with blue indicating negative results and orange indicating positive results. NTC represents no-viral DNA control.

To simplify the manipulation procedure of the two-pot RPA-CRISPR/Cas12a-PCV3 detection method and reduce the risk of secondary operations or aerosol contamination [22], the two-pot detection method was further optimized. We combined the RPA amplification step and the CRISPR/Cas12a-PCV3 detection step into one tube [23], in which the reagents for RPA amplification and CRISPR/Cas12a-PCV3 detection were added to the bottom and cap of the tube, respectively (Figure 5A). After RPA amplification, the reagents were rotated to initiate CRISPR/Cas12a detection. The results showed that the minimum detection limit of this one-pot detection method could reach 2 × 10^1^ copies/µL (Figure 5B,C). To determine a more accurate detection limit, the refined viral DNA (concentrations 2 × 10^1^, 1 × 10^1^, 0.5 × 10^1^, and 0.25 × 10^0^ copies/µL) were further detected. The results showed that the minimum detection limit of the one-pot detection method was 0.5 × 10^1^ copies/µL (5 copies/µL) (Figure 5D,E). Subsequently, 25 clinical samples were analyzed using the one-pot method to evaluate their performance. The results showed that 18 out of 25 clinical samples were positive, and the remaining samples were negative (Figure 6A,B), suggesting that the one-pot RPA-CRISPR/Cas12a-PCV3 detection method is feasible.

## 4. Discussion

PCV3 infections have been reported worldwide, causing huge economic losses to the swine industry. The early diagnosis of various pathogens plays an important role in the prevention and control of related diseases. Nucleic acids, which are the most conventional detection targets, have been used for the diagnosis and detection of various pathogens, which has prompted nucleic acid amplification, such as PCR-related techniques, to become a sensitive and specific method [10,11]. However, these techniques have limited application owing to expensive instrumentation and complex operation procedures. Thus, a better method that overcomes these shortcomings is urgently required for pathogen detection. In this study, we developed a nucleic acid detection method targeting the PCV3 Cap gene, the RPA-CRISPR/Cas12a-PCV3 detection method, by combining the RPA and CRISPR/Cas12a techniques, in which crRNA activates Cas12a endonuclease activity by recognizing specific target sequences, followed by cleaving an ssDNA-FQ probe to produce a fluorescent signal for PCV3 diagnosis. 

To date, some isothermal amplification methods, such as loop-mediated isothermal amplification (LAMP) [24], enzymatic recombinase amplification (ERA) [25], and RPA [26], have been developed as improved methods over conventional PCR methods, based on their simplicity, rapidity, and low cost. However, the emergence of nonspecific amplification signals in these detection methods limits their application [27]. Recently, the combination of crRNA-mediated CRISPR/Cas endonuclease targeting nucleic acid detection techniques has enhanced the specificity of isothermal amplification methods [28,29]. This high specificity is attributed to the specific recognition of PAM sites by the Cas protein in the presence of crRNA [30]. Cas12a performs nonspecific cleavage of ssDNA by recognizing specific dsDNA sequences, which is suitable for the detection of dsDNA viruses such as PCV3 and ASFV. 

The CRISPR/Cas12a detection conditions, such as Cas12a endonuclease activity, probe concentration, Cas12a/crRNA ratio, and detection sensitivity of the Cas12 enzyme, were optimized according to the results of the fluorescence signal (Figures S1 and S3). The Cas12a enzyme possesses only low detection sensitivity without pre-amplification because of its weak cleavage activity [12,31]. Our results confirmed the low sensitivity of Cas12a and showed that the detection sensitivity of the Cas12a enzyme itself was only 2 × 10^9^ copies/µL viral DNA (Figure S3B,C), which verified the character of the low sensitivity of Cas12a activity. Thus, CRISPR/Cas12a detection must be combined with isothermal amplification to enhance sensitivity. We established a two-pot RPA-CRISPR/Cas12a-PCV3 detection method (Figure 1), which not only shortens the detection time but also improves detection sensitivity, with a minimum detection limit of two copies/µL of viral DNA (Figure 2). The detection sensitivity of this method is superior to that of previously reported methods, such as the TagMan-based real-time PCR [32], LAMP [33], and RPA assays [34]. The specificity of this method did not show any cross-positive reactions targeting other viruses (Figure 3B–E), which is important for clinical detection. Subsequently, the coincidence rate of the 25 clinical samples detected between the two-pot RPA-CRISPR/Cas12a-PCV3 detection method and the previously reported qPCR method was 100% (Figure 4), and all positive samples were further validated by sequencing, suggesting the reliability and utility of the two-pot method. We further integrated the amplification and detection steps into a single tube to simplify the operation and reduce pollution (Figure 5). Our established one-pot detection method reached a sensitivity limit of five copies/µL viral DNA, and its detection results of the clinical samples were completely consistent with the two-pot method (Figure 6), indicating that the one-pot method is reliable and suitable for a clinical test.

## 5. Conclusions

In summary, we successfully developed one- and two-pot RPA-CRISPR/Cas12a-PCV3 detection methods that could detect PCV3 within 30 min at a minimum detection limit of five and two copies of viral DNA, respectively. These new methods with high sensitivity, specificity, and short assay times provide advantages for virus detection and are suitable for the prevention of the occurrence and spread of PCV3 at an early stage.

## Figures and Tables

**Table 1:** Comparison between two-pot RPA-CRISPR/Cas12a and qPCR detections for clinical samples.

Sample Types	Sample Numbers	RPA-CRISPR/Cas12a	qPCR
Spleen	6	6/6	6/6
Lung	7	5/7	5/7
Kidney	2	1/2	1/2
Submandibular lymph node	3	3/3	3/3
Inguinal lymph node	7	3/7	3/7
Positive rates		72%	72%

## Data Availability

The original contributions presented in the study are included in the article/supplementary material, further inquiries can be directed to the corresponding author.

## Supplementary Materials

The following supporting information can be downloaded at: https://www.mdpi.com/article/10.3390/ani14172527/s1, Figure S1. The expression and purification of Cas12a proteins. Figure S2. The validation of three crRNAs and optimization of the Cas12a/crRNA concentration in CRISPR/Cas12a reaction. Figure S3. The validation of RPA primers and the sensitivity determination of the CRISPR/Cas12a reaction. Table S1. Primers, crRNA, and probe in this study.

## Abbreviations

PCV3, Porcine circovirus type 3; qPCR, real time quantitative PCR; CRISPR, clustered regularly interspaced short palindromic repeats; crRNA, CRISPR RNA; PAM, protospacer adjacent motif; dsDNA, double-stranded DNA; ssDNA, single-stranded DNA; RPA, recombinant enzyme polymerase amplification; PCV, porcine circovirus; PRV, pseudorabies virus; SVA, Senecavirus A; PRRSV, porcine reproductive and respiratory syndrome virus; CSFV, classical swine fever virus.
