# Supplementary material for: Recombinant Polymerase Amplification Coupled with CRISPR/Cas12a Detection System for Rapid Visual Detection of Porcine Circovirus 3
Source: Animals (Basel). 2024 Aug 30;14(17):2527. doi: 10.3390/ani14172527 (PMC11393983; doi:10.3390/ani14172527)
Supplement: Supplementary file 1 [file animals-14-02527-s001.zip › animals-3152930-supplementary.pdf]

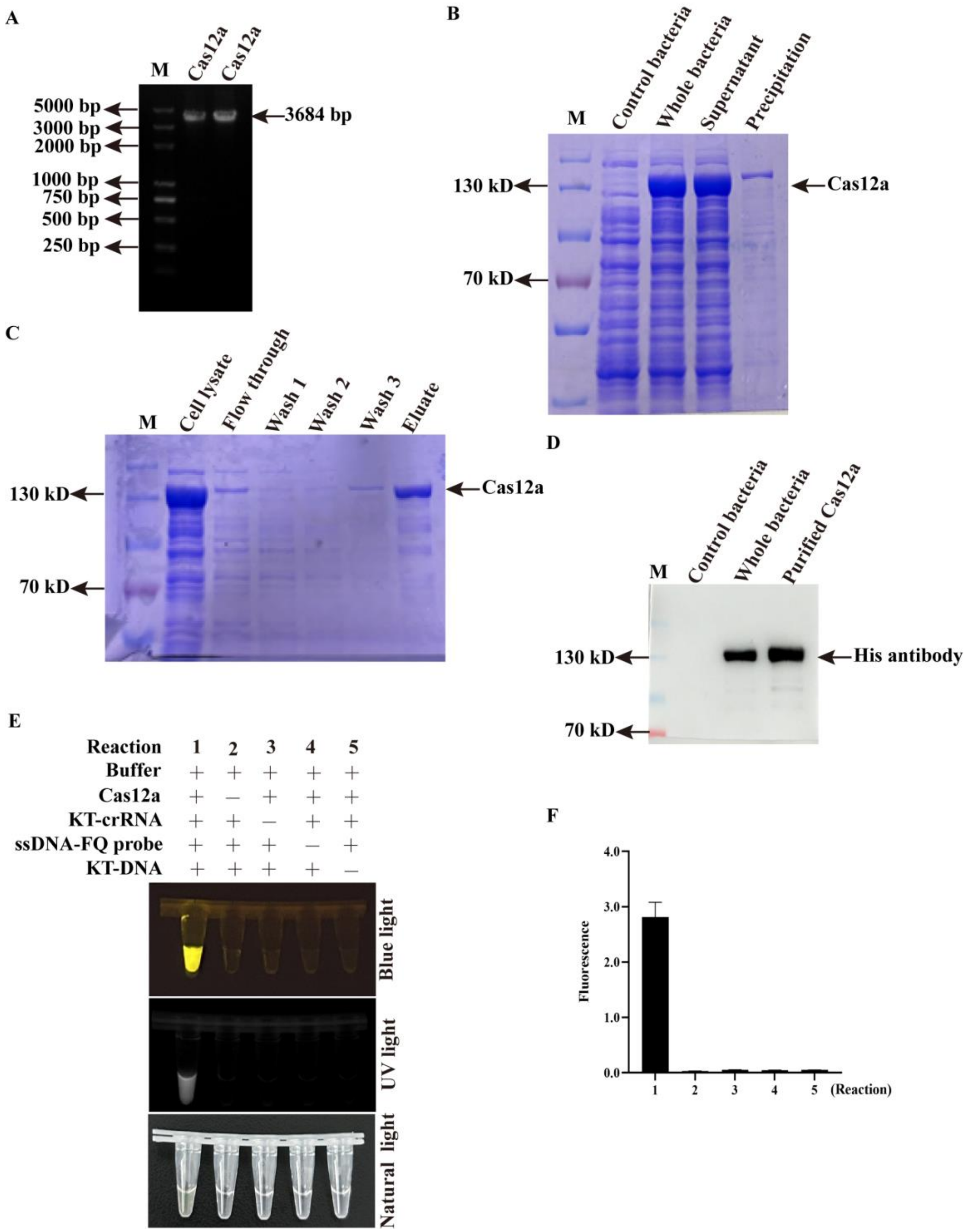

Figure S1. The expression and purification of Cas12a proteins. (**A**) The construction of recombinant pET28a-Cas12a plasmid. (**B**) The expression of the Cas12a proteins. (**C**) The purification of Cas12a proteins. D, The detection of purified Cas12a proteins by western blotting. (**E**,**F**) The measurement of CRISPR/Cas12a reaction in the presence of KT-DNA and KT-crRNA by blue light, UV light, natural light (**E**), and fluorescence signal (**F**). The data are expressed from three independent experiments.

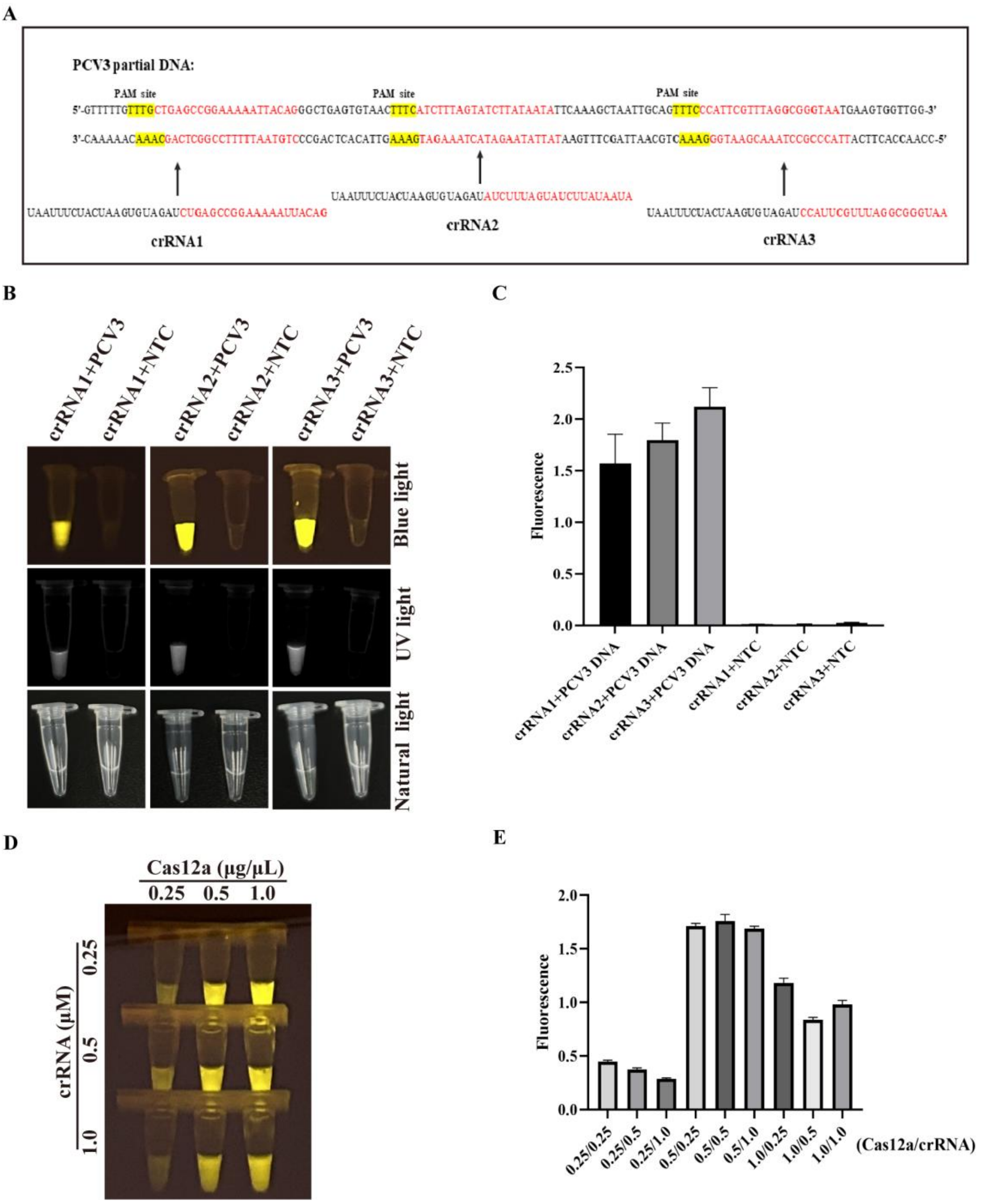

Figure S2 The validation of three crRNAs and optimization of the Cas12a/crRNA concentration in CRISPR/Cas12a reaction. (**A**) Schematic diagram of targeting sites for three crRNAs. (**B,C**) Verification of three crRNAs for PCV3 DNA by blue light, UV light, natural light (B), and fluorescence signal (**C**). (**D,E**) The measurement of CRISPR/Cas12a reaction with various Cas12a/crRNA concentrations by blue light (**D**) and fluorescence signal (**E**). NTC represents no-PCV3 DNA control. The data are expressed from three independent experiments.

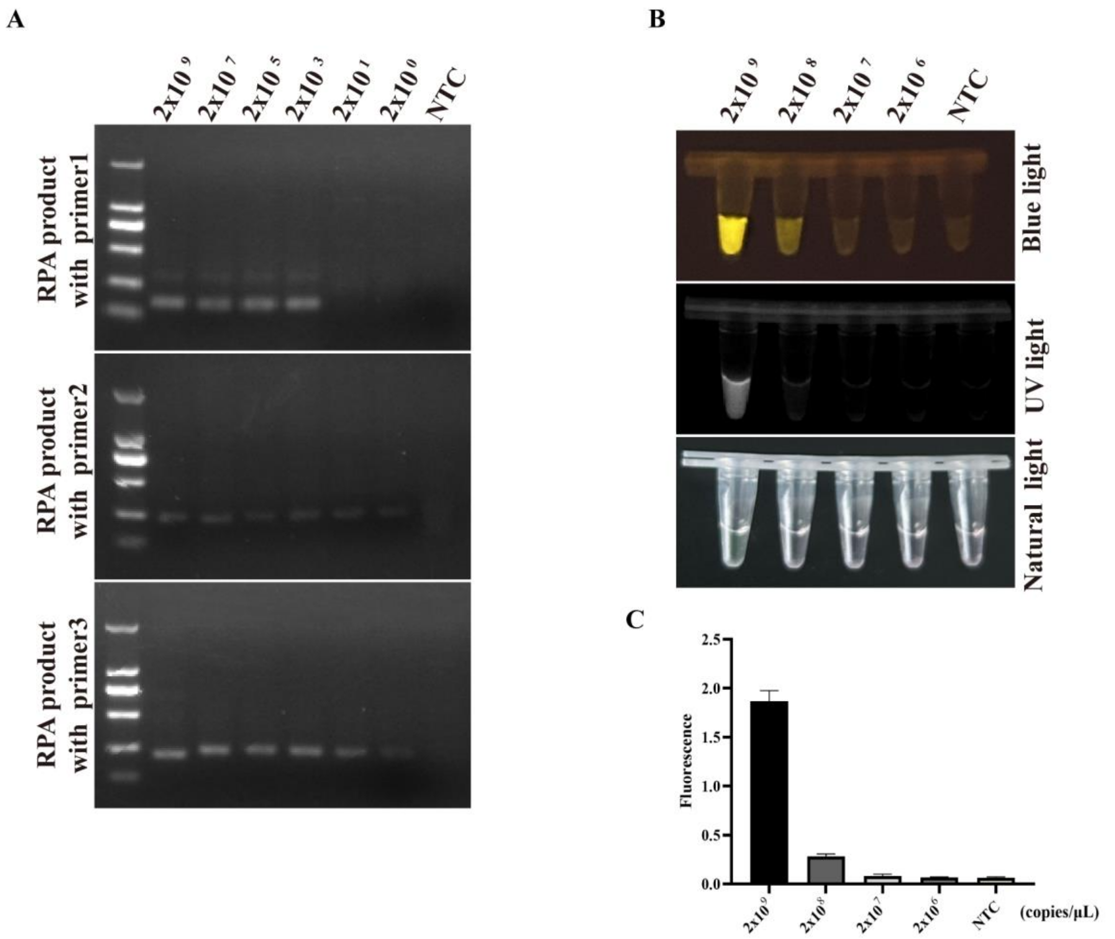

Figure S3 The validation of RPA primers and the sensitivity determination of the CRISPR/Cas12a reaction. (**A**) Amplification efficiency of various RPA primers. (**B**,**C**) The sensitivity measurement of the CRISPR/Cas12a reaction targeting PCV3 DNA (concentrations from 2×10^9 to 2×10^6 copies/μL) by blue light, UV light, natural light (**B**), and fluorescence signal (**C**). NTC represents no-PCV3 DNA control. The data are expressed from three independent experiments.

Table S1. Primers, crRNA, and probe in this study

**Name**	**Sequence (5'-3')**
pET28a-Cas12a-F	GCGGGATCCATGAGCAAGCTGGAGAAGTTTAC
pET28a-Cas12a-R	ATGTCGACTGTGCTTCACGCTGGTCTGGGC
crRNA1	UAAUUUCUACUAAGUGUAGAUCUGAGCCGGAAAAAUUACAG
crRNA2	UAAUUUCUACUAAGUGUAGAUAUCUUUAGUAUCUUAUAAUA
crRNA3	UAAUUUCUACUAAGUGUAGAUCCAUUCGUUUAGGCGGGUAA
RPA-F1	CTCCACCATGAACGTCATTTCCGTTGGAAC
RPA-R1	TTACAGGGCTGAGTGTAACTTTCATCTTTAG
RPA-F2	CTCCACCATGAACGTCATTTCCGTTGGAAC
RPA-R2	CTATGGCTGTGTGCCCGAACATAGTTTTTGTTTGC
RPA-F3	CTCCACCATGAACGTCATTTCCGTTGGAAC
RPA-R3	TCTTGGAGCCAAGTGTTTGTGGTCCAGGCGCCG
ssDNA-FQ probe	FAM-TTATT-BHQ1
KT-DNA-F	GCTCGAGTTATGAATGCGCAAGTTCAGC
KT-DNA-R	TAGTCGACATGGACACTGAAACGTCTCCAC
KT-crRNA	UAAUUUCUACUAAGUGUAGAUGGUAUGCUCGACUUGCAGUC
PCV1-F	GAGGGTAGAACTCCTCACCT

PCV1-R	AGCTGGGACAGCAGTTGAG
PCV2-F	ATGCCCAGCAAGAAGAGTGG
PCV2-R	CACTTTCAAAAGTTCAGCCA
PCV4-F	ATTCTCTGAATTGCACATACAGA
PCV4-R	TGTGGCCCCGGGCCAGTAGGCGG
PRRSV-F	GGCTTGGTTGTTACTGAGACAG
PRRSV-R	GAGGGTACAAGGGGTAACAGTT
PRV-F	GCTCAGTCGTCGTCCTGGGTGA
PRV-R	CTGCCGTGCATGCACAAGTTC
SVV-F	ATGATTTTGATTCCCGCGGCAAT
SVV-R	GTCCACAAAATGTCAGAGTGAT
PEDV-F	GTTTGAAACCAGTAACTGTCGGCT
PEDV-R	AACACCAGTGCCAGCACCAGC
CSFV-F	ATGTTTTCTCTTCAAGACATCTGT
CSFV-R	TTACTCACATTTCAATTGAAGTGCT
qPCR-KTF	GCTACGAGTGTCCTGAAGAT
qPCR-KTR	GCCTCCACACTCCACAATAG
